# Impact of Antibiotics on Efficacy of Cry Toxins Produced in Two Different Genetically Modified Bt Maize Varieties in Two Lepidopteran Herbivore Species, *Ostrinia nubilalis* and *Spodoptera littoralis*

**DOI:** 10.3390/toxins10120489

**Published:** 2018-11-23

**Authors:** Angelika Hilbeck, Nicolas Defarge, Thomas Bøhn, Michelle Krautter, Constanze Conradin, Caroline Amiel, Jean-Michel Panoff, Miluse Trtikova

**Affiliations:** 1 Swiss Federal Institute of Technology, Institute of Integrative Biology IBZ, Universitätstrasse 16, CH-8092 Zurich, Switzerland; nicolas.defarge@env.ethz.ch (N.D.); michelle.krautter@outlook.com (M.K.); constanze.conradin@gmail.com (C.C.); miluse.trtikova@env.ethz.ch (M.T.); 2 Institute of Marine Research, Department Tromsø, Hjalmar Johansens Gate 14, 9296 Tromsø, Norway; thomas.bohn@hi.no; 3 Biosfera Val Müstair, Center da Biosfera, 7532 Tschierv, Switzerland; 4 Normandie University, UNICAEN, EA4651 ABTE, Boulevard Maréchal Juin, CEDEX 5, CS 14032 Caen, France; caroline.amiel@unicaen.fr (C.A.); jean-michel.panoff@unicaen.fr (J.-M.P.)

**Keywords:** ecotoxicology, *Bacillus thuringiensis*, Cry toxins, antibiotics, nontarget organisms, lepidoptera

## Abstract

The insecticidal crystal proteins from *Bacillus thuringiensis* (Bt) are widely-used biopesticides that are used both as Bt spore-crystal preparations in sprayable formulations and as activated toxins in genetically modified (GM) plants. Models for their modes of action have been proposed but many issues remain unresolved. Among those is the role of commensal gut bacteria in target insect death: previous studies showed that antibiotics attenuate the toxicity of Bt sprays. We tested whether antibiotics interfere with the effects of GM plant-produced Bt toxins in larvae of two Lepidopteran species, the European corn borer *Ostrinia nubilalis* and the cotton leafworm *Spodoptera littoralis*. The larvae were reared on artificial diet with or without antibiotics and, thereafter, fed two varieties of Bt GM maize in comparison to conventional non-Bt maize leaves sprayed with antibiotic solution and/or with a Bt formulation. Antibiotics significantly reduced or delayed the toxicity of Cry toxins, although to a lesser extent than previously reported for Bt-sprays. This supports the hypothesis that Cry toxins induce mortality by themselves in the absence of Bt bacteria and spores, and of commensal gut bacteria. However, larvae that were not treated with antibiotics died faster and at a higher rate which was further compounded by plant variety and species sensitivity. These findings support a hypothesis that toxicemia alone can inflict significant mortality. However, in the absence of antibiotics, the gut bacteria likely enhance the Cry toxin effect by inflicting, additionally, bacterial septicemia. This has important implications in field situations where antibiotic substances are present—e.g., from manure of animals from conventional production systems—and for ecotoxicological testing schemes of Bt toxins and nontarget organisms that are often using artificial diets enriched with high concentrations of antibiotics.

## 1. Introduction

The bacterial toxins from *Bacillus thuringiensis* (Bt) have become the most widely used microbial insecticides in the world. Transgenic DNA from the Bt bacterium coding for crystalline (Cry) toxins (mainly from the classes Cry1, Cry2, Cry3) have been genetically engineered into a number of globally grown and industrially traded commodity crops including maize, cotton, soybeans [1,2,3]. In parallel to the increasing production areas of these GM Bt crops, mainly in North- and South America [4], the controversy around the mode(s) of action of Cry toxins has also increased [5,6] and there is today less certainty about the precise steps leading to the death in targeted insect pests than there was at the time of first introduction of GM Bt crops over 20 years ago. Also, the range of affected organisms, beyond and above the target pest species, is debated within differing narratives of ‘specificity’ [5], especially in context of GM plant-produced Cry toxins which differ from those produced by bacteria [7].

Bt proteins as produced by *B. thuringiensis* bacteria are ingested as inactive crystalline (Cry) proteins [6]. At least for the most studied class of Bt toxins, the Cry1 class, these crystalline proteins have to be solubilized in the insect gut and undergo further biochemical cleavage to form the active Cry toxins. According to the ‘classical model’, the Cry toxin then binds to certain receptors such as cadherins located in the midgut epithelium and directly forms pores in the cell membrane provoking osmotic shock and resulting in cell lysis. The impairment of the midgut epithelium allows gut bacteria access to the hemolymph where they multiply, leading to fatal septicemia [2,6,8]. However, over the past 10 years, two more proposed models are debated: one, the sequential binding model, complementing the classical pore forming model by proposing additional steps such as cleavage of an alpha-helix and oligomerization prior to the pore formation, while the other challenges the formation of pores, or at least its absolute requirement in the process of cell lysis. Instead, it proposes a ‘signal transduction model’ where binding of Cry toxin monomers to cadherin activates intracellular cell death mechanisms [9,10,11]. The upregulation of caspase-4 transcripts [12] observed in *Ostrinia nubilalis* exposed to Cry1Ab may partly corroborate this pathway. Nonetheless, this model has been questioned by Vachon et al. [6] and also by Soberon et al. [13] who developed the competing ‘sequential binding model’. Yet others have suggested a combination of the ‘signal transduction model’ and the pore formation model to be at work [14,15,16]. As stated by Adang et al. [17]: “it is plausible that intracellular cell death may occur in the presence of lower Cry toxin concentrations, while higher toxin concentrations would promote increased toxin insertion and formation of pores”. Although all models are contested to some degree, several authors seem to agree that, still today, critical aspects of the mode of action of the complex Bt toxins are ‘poorly understood’ [6,18] and that “further work to establish functional connections between pore formation and intracellular signaling for cytotoxicity are needed to clarify the molecular events resulting in midgut cell death by Cry toxins” [17].

Meanwhile, Broderick and colleagues added a whole new layer of complexity to this controversy when suggesting that Cry toxins may, in fact, not even be the directly lethal agent but rather only a helper for other lethal agents. Indigenous gut bacteria switching from commensal to pathogen are suggested to actually kill Bt-susceptible insects [19,20,21]. The authors found that toxicity of the Bt bacterium-based insecticide ‘Dipel’ (spores and Cry toxins; Valent BioSciences) on *Manduca sexta* (L.), *Vanessa cardui* (L.), *Pieris rapae* (L.), or *Lymantria dispar* (L.) was significantly reduced when larval gut bacteria were eliminated below detectable levels by oral administration of antibiotics. Additionally, they showed that the re-establishment of an *Enterobacter sp*. in aseptic rendered *Lymantria dispar* larvae restored the insecticidal activity of Bt to nearly that of larvae not treated with antibiotics. These findings led to the suggestion that the actual deadly septicemia is not caused by Bt itself but rather by gut bacteria entering and multiplying in the larval hemolymph. Hence, the authors postulated that while the *B. thuringiensis* produced Cry toxins induce pore formation and subsequent impairment of the midgut epithelium of susceptible larvae which allows gut bacteria to enter the hemocoel, it is these gut bacteria, not Bt bacteria nor its toxins, that ultimately kill the host larvae via septicemia [19,20]. They found that Bt bacteria could not sufficiently multiply in hemocoel while gut bacteria could. Hence, *Enterococcus faecalis*, which are commensal bacteria in an intact insect gut, can become and act as a pathogen when invading the hemocoel. A process Mason and colleagues [21] studied in detail and referred to as the ‘commensal-to-pathogen’ switch. Thereby, rendering Bt possibly an ‘impotent pathogen’ unable to kill the host organism on its own.

Raymond, et al. [22,23] and Johnston and Crickmore [24] challenged these findings and postulated that no microbial gut community is required to kill the diamondback moth (*Plutella xylostella*) with *B. thuringiensis*. Raymond et al. [23] state that *B. thuringiensis* is “a true pathogen in its own right”. Both Raymond et al. [22] and Johnson and Crickmore [24] postulate that the effect observed by Broderick et al. [19,20] is caused by the direct antibiotic effect on the *B. thuringiensis* bacteria, alongside eliminating the gut bacteria as well. They further hypothesized that *B. thuringiensis* synergizes facultatively pathogenic gut bacteria when all, *B. thuringiensis* plus gut bacteria, enter the hemocoel after pore formation induced by Cry toxins and subsequent cell lysis by osmotic shock. Furthermore, they showed that *B. thuringiensis* bacteria could grow rapidly in plasma from hemocoel of *Manduca sexta*, *Spodoptera litura*, and coleopteran *Tenebrio molitor* larvae and speculated that prior immunization of larvae with *E. coli* elicits an increase in immunity to subsequent *B. thuringiensis* infections. Recently, Caccia et al. [25] published data confirming that gut bacteria are critical in killing larvae of *Spodoptera littoralis* but further suggested that this process is modulated by the immune response and immune defense capacity of the larvae against bacteria entering the hemocoel.

In conclusion, the key issue of the controversy seems to be whether septicemia (by *B. thuringiensis* bacteria and/or commensal gut bacteria) or toxicemia (by the Cry toxins) are the prime killing agents alone or in conjunction. Experiments with GM plants expressing solely activated forms of Cry toxins and none of the other contested components, like *B. thuringiensis* bacteria, spores or crystals, will add complementary data still missing in this contentious debate. Although Mason et al. [21] administered Cry toxins from a commercial formulation of GM *Pseudomonas fluorescens* bacteria, no studies were carried out to date with Cry toxins produced in GM plants. With GM Bt crops, the role of any *B. thuringiensis* cells or spores, or those of other GM Bt-expressing microbes (e.g., *P. fluorescens*, *E. coli*) is a non-issue, the mode of action of GM plant produced Cry toxins hinges solely on their biochemical characteristics. Any effect of added antibiotics would to a large extent, if not exclusively, be limited to interfering with the commensal gut bacteria in the test species. Using GM plants expressing Cry toxin is also interesting since the biochemical form of Cry toxins produced by GM plants differs significantly from those produced in GM bacteria [7].

Furthermore, understanding the role of antibiotics on efficacy of Cry toxins has ramifications regarding ecotoxicity testing for non-target organisms. In Europe, and in countries that are signatories to the Cartagena Protocol on Biosafety, GM plants can only be placed on the market after regulatory approval. Within this regulatory approval process a risk assessment has to be carried out that, for GM Bt plants, require basic ecotoxicity testing with some non-target organisms for potential adverse effects of the Bt toxins expressed in these plants [26]. Typically, these organisms are tested using the purified Cry toxin extracted from GM microorganisms, not GM Bt plants. These purified, isolated Cry toxins of bacterial origin are often then administered to the test organisms by mixing them into an artificial diet. Importantly, these artificial diets for ecotoxicity testing or mass rearing of insect larvae are often reported to contain antibiotic substances to prevent decay of the diets [27]. In recent years, a number of studies were published specifically proposing certain artificial diet recipes for testing of Cry toxin effects on non-target beneficial insects within the scope of biosafety and risk assessment of GM Bt plants [28,29,30]—all of them contained high amounts of antibiotics.

In our study presented here, we investigated whether the administration of antibiotics known to significantly decrease or eliminate gut bacteria could modulate the toxicity of GM Bt plants on two lepidopteran species, one being highly susceptible (*Ostrinia nubilalis*) and one being only moderately susceptible (*Spodoptera littoralis*) to Bt toxins. Furthermore, these tests were done with two different GM Bt maize varieties expressing the same transgene event, MON810, that codes for the Cry1Ab toxin, and the closest available conventional non-GM comparator maize. We further compared this to a commercial *B. thuringiensis*-based spore formulation (Delfin).

## 2. Results

### 2.1. Effects of Antibiotics on Midgut Bacterial Strains

**Figure 1:**
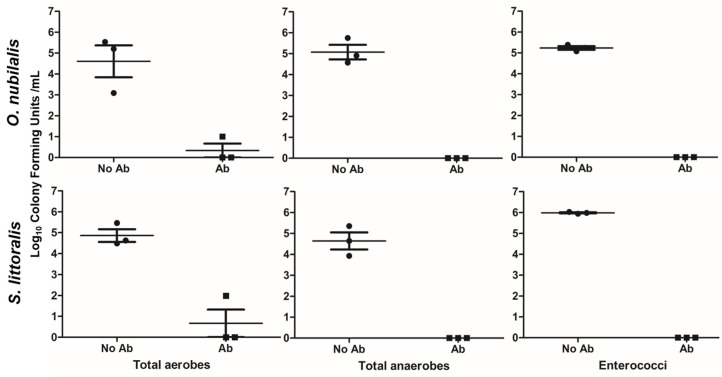
Pretreatment with antibiotics drastically reduced the cultivable midgut microbes of *S. littoralis* and *O. nubilalis* in selective media. A cocktail of antibiotics was administered 3 or 5 days to *S. littoralis* and *O. nubilalis*, respectively. Data are shown as mean of the Log10 CFU (colony forming units) ± standard error of the mean (SEM).

The pre-assay antibiotics treatment did not affect the larval survival. However, it was efficient to significantly (at least *p* < 0.01 in paired *t*-tests) reduce the cultivable midgut bacterial strains, even below detectable levels in two of the three selective media (Figure 1).

### 2.2. Impacts of GM Bt Maize with or without Antibiotic Treatments

#### 2.2.1. Mortality of *O. nubilalis* and *S. littoralis* Larvae Fed GM Bt Maize Varieties without Antibiotics

**Figure 2:**
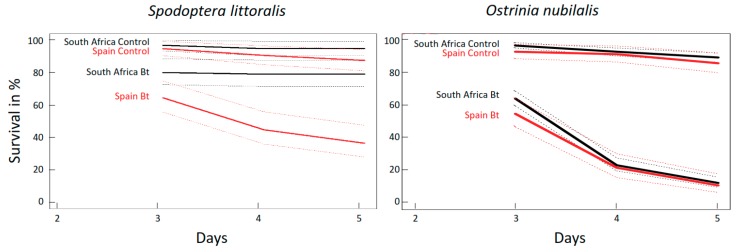
Effects of Bt and control maize varieties (no antibiotic treatment) from South Africa and Spain, on larval survival in *S. littoralis* and *O. nubilalis*. All plant varieties were grown under identical laboratory conditions.

For both insect species, survival was significantly lower for larvae that fed on Bt maize than on non-Bt maize, regardless of the varietal background (*p* < 0.001, coxph test) (Figure 2).

However, for Bt maize fed *S. littoralis*, the moderately susceptible nontarget pest, maize variety (South African vs. Spanish) influenced survival significantly. Larvae fed South African Bt maize had much higher survival (about 80%) than larvae fed Spanish Bt maize (below 40%) (Figure 2, left panel), the differences were highly significant (*p* < 0.001, coxph test). A similar but weaker (non-significant) trend was seen for *S. littoralis* fed non-Bt control maize (*p* = 0.09, coxph test).

Survival of *O. nubilalis*, the highly susceptible target pest species, was much lower on both Bt maize varieties. However, 15–20% of the larvae on both varieties were still alive after five days (Figure 2, right panel). In contrast, *O. nubilalis* had a high survival rate on both non-Bt control maize varieties.

#### 2.2.2. Effects of Antibiotics (AB) on Survival of *O. nubilalis* and *S. littoralis* Larvae Fed GM Bt Maize Varieties

In both insect species, the AB treatments did not affect the survival of larvae fed non-Bt control maize (data not shown). However, the two insect species exhibited different responses to the combined effects of Bt maize variety and AB treatment. Therefore, we present the data for each insect species separately below.

**Figure 3:**
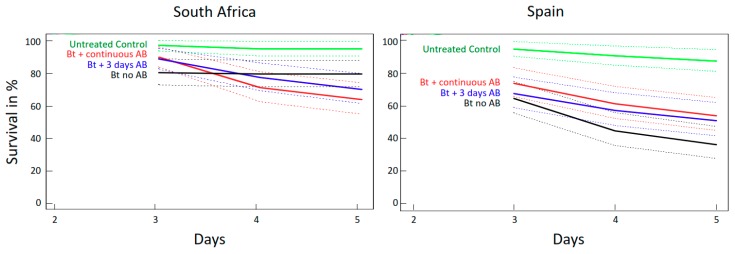
Survival curves with 95% CI of *S. littoralis* fed Bt maize from South Africa and Spain, with no AB treatment, three days’ pretreatment with AB, or continuous AB treatment, respectively. The survival of larvae fed untreated (no AB) non-Bt maize is included for comparison.

Regardless of Bt maize variety, there were no statistically significant differences between survival of *S. littoralis* larvae raised on Bt maize that were pretreated for only 3 days prior to the bioassay (Figure 3, blue lines) or treated continuously with antibiotics (Figure 3, red lines) (*p* = 0.381 and 0.573 coxph test, respectively). Therefore, we combined these two groups for statistical analyses and tested them against the *S. littoralis* larvae fed untreated (no AB) Bt maize (Figure 3, black lines).

*S. littoralis* larvae raised on South African Bt maize treated with AB had similar high survival rates (65–80%) than those raised on untreated (no AB) Bt maize (Figure 3 left panel, *p* = 0.07, coxph test). *S. littoralis* larvae raised on Spanish Bt maize treated with AB had significantly higher survival than larvae raised on untreated Bt maize (Figure 3 right panel, *p* = 0.014, coxph test).

**Figure 4:**
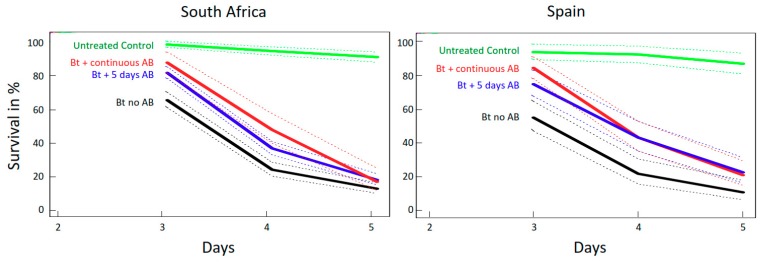
Survival curves with 95% CI of *O. nubilalis* fed Bt maize from South Africa and Spain with, respectively, no AB treatment, five days’ pretreatment with AB, or continuous AB treatment. The survival of larvae fed untreated (no AB) non-Bt maize is included for comparison.

Regardless of Bt maize variety, all *O. nubilalis* larvae fed Bt maize with AB had a significantly higher survival than those raised on Bt maize without AB treatment (Figure 4; *p* < 0.001, coxph test for both South African and Spanish Bt-maize material). Although there were only small differences in final survival rates, larvae treated with AB died later. On day 4, on both Bt maize varieties, almost twice the number of larvae were still alive when treated with AB than on the untreated Bt maize (Figure 4).

The group fed Bt maize treated continuously with AB had marginally higher survival and showed slightly delayed mortality, compared to the groups only pretreated with AB for five days (Figure 4).

#### 2.2.3. Effects of Antibiotics (AB) on Weight Gain of *S. littoralis* Larvae Fed GM Bt Maize Varieties

**Figure 5:**
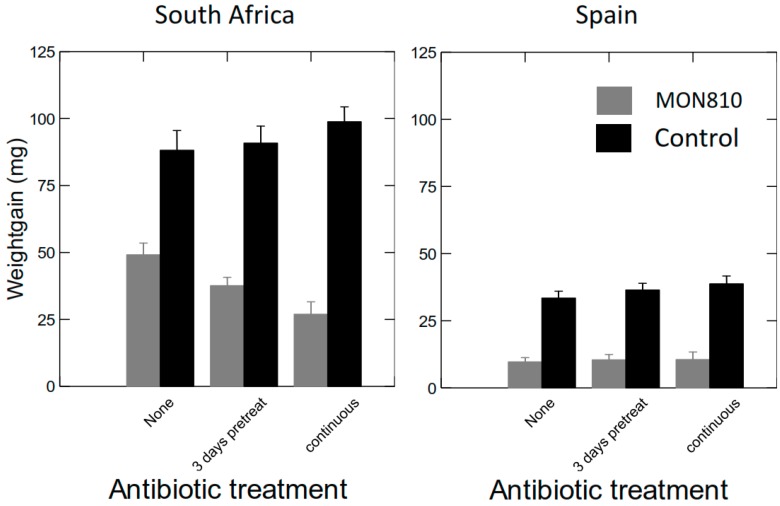
Weight gain with SE of *S. littoralis* fed Bt and non-Bt Control maize from South Africa (**left panel**) or Spain (**right panel**) with different AB treatments.

On South African non-Bt control maize, the weight gain was on average more than twice as high than in larvae fed Bt maize (Figure 5 left panel) (ANCOVA, *p* < 0.001). On non-Bt maize, the effect of the AB treatments did not cause significant differences in weight gain of *S. littoralis* larvae (ANCOVA, TUKEYHSD, *p* > 0.07). However, on Bt maize, weight gain was somewhat reduced when larvae were treated with AB (ANCOVA, TUKEYHSD, *p* < 0.001). (Figure 5 left panel).

On Spanish maize, the weight gain was more than three times as high in larvae fed non-Bt control maize than when fed Bt maize (Figure 5 right panel) (ANCOVA, *p* < 0.001). However, on the Spanish maize varieties, no significant differences in weight gains due to the AB treatments were found, neither on the Bt nor on the non-Bt control maize (ANCOVA, TUKEYHSD, *p* > 0.94 and *p* > 0.32, respectively). Compared to the weight gain on South African maize varieties (Figure 5 left panel), regardless whether with or without Bt, weight gains were 3–4 times less on the Spanish maize varieties (Figure 5, right panel), confirming the observation of somewhat lower suitability of the Spanish maize varieties for larval survival (Figure 2).

### 2.3. Effects of Antibiotics on Survival of *O. nubilalis* and *S. littoralis* Larvae Fed Bt-Sprayed Non-GM Maize

**Figure 6:**
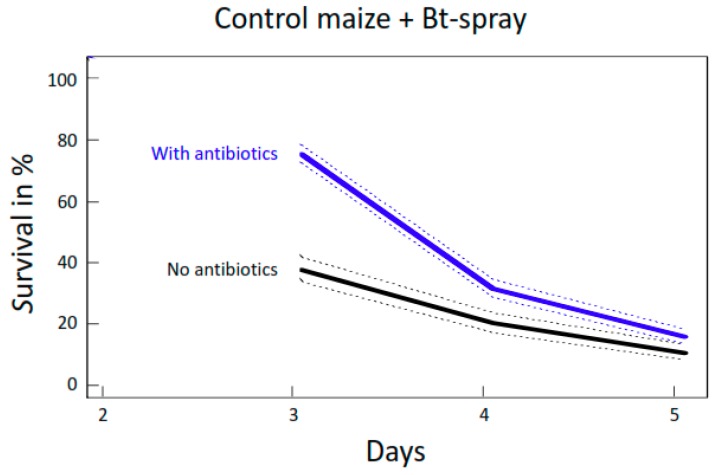
Survival curves with 95% CI of *O. nubilalis* fed Bt-sprayed (Delfin) control maize from South Africa with or without antibiotic treatment.

When raised on Bt-sprayed control maize (non-Bt South African variety), survival was significantly greater in *O. nubilalis* larvae until day 4 when they were pre-treated with AB for five days prior to the bioassay compared to no AB treatment (Figure 6; *p* < 0.001, coxph test). Interestingly, however, survival did not exceed 20%. Without AB, most larvae died quickly. On day 3, only 40% of the larvae on Delfin-sprayed maize without AB were still alive, in contrast to 80% survival for larvae fed AB treated Delfin-sprayed maize (Figure 6).

## 3. Discussion

Oral administration of antibiotics in most cases significantly reduced and delayed the toxicity of Cry toxins regardless whether they were of plant or microbial origin, although to a much lesser extent than reported by others. Furthermore, there was a significant plant variety effect that was most pronounced in the less susceptible species, *S. littoralis*, with the South African GM Bt maize variety being much less effective than the Spanish Bt maize variety. Consequently, for *S. littoralis*, the modulating effect of the antibiotic treatment was less pronounced on the South African Bt maize than on the Spanish Bt maize. These differences in *S. littoralis* survival rates could not be explained by different Cry toxin concentrations, as we found in parallel studies with other plants of the same varieties raised under identical conditions that both Bt maize varieties contained similar concentrations of Cry toxin (unpublished data: South African Bt maize: mean 46.4 µg/g dry weight, range 25.6–99.8; Spanish Bt maize: mean 44.94 µg/g dry weight, range 31.6–58.7—ELISA methodology as published by Trtikova et al. [31]).

For *O. nubilalis*, although the antibiotic treatment delayed mortality, overall survival rates ended up similar on both Bt maize varieties although survival was somewhat higher in insects treated with antibiotics. However, the expectation that there would be no survivors on a Cry1Ab maize (at least those untreated with antibiotics) was not met either, although these varieties are considered high-expressing Bt maize events for *O. nubilalis* (e.g., [32,33]). This might be owed to the fact that the larvae were older already and the Bt toxin affects most severely young larvae.

Our overall results indicate that Cry toxins were the main killing agent in the susceptible *O. nubilalis* larvae, in particular when the larvae were treated with antibiotics that have been demonstrated to severely decrease or eliminate commensal gut bacteria (Figure 1). However, larvae that were not treated with antibiotics died faster and at a higher rate which was further compounded by plant variety and species sensitivity. These findings support a hypothesis that toxicemia alone, probably induced by Cry toxin-caused trauma in insect midgut epithelial tissue, can inflict significant mortality. In the absence of antibiotics, the gut bacteria likely enhanced the Cry toxin effect by inflicting, additionally, bacterial septicemia.

### 3.1. Contextualizing with Previous Studies

In Table 1, we summarize the various hypotheses, protocols and data obtained by the various research groups discussed here. All used bacteria-produced Cry proteins, mostly from various commercial formulations but some also collected proteins from *B. thuringiensis* or purified proteins from laboratory bacteria colonies. In formulations, Cry proteins are typically present in their crystalline form and/or as protoxins. However, for all bacteria-produced Cry toxins, more or less uncertainty remains regarding the possible interaction role of cells or spores remaining from these microbes, be these *B. thuringiensis* itself or other GM bacteria like *E. coli* or *P. fluorescens*. Furthermore, all Cry proteins produced by these various bacteria are presumably initially inactive (crystals and/or protoxins) and require activation through sequential cleavage into smaller toxic fragments that are presumed to be the prime causal agent for the formation of pores, triggering further degrading processes that ultimately lead to the breaching of the gut wall into the hemocoel of the insect. Without this breaching, obviously, no gut bacteria can enter the hemocoel.

In all the listed studies in Table 1, the administration of broad-spectrum antibiotics was used as proxy for presence and absence of gut microbiota, which had been documented in some of the studies by directly testing gut or hemocoel content to detect at least cultivable bacteria. While Cry toxins in GM crops have been tangentially mentioned by some authors (e.g., in [18,19,34]), to our knowledge, we are the first to study this issue experimentally.

Our data show that antibiotics modulate significantly the onset of the impact and to a lesser extent also the severity/efficacy of Cry toxins. Some of the previous publications have also reported about substantial delays in the onset of mortality when treated with antibiotics (e.g., [24]). Notably, however, most of the studies have reported much stronger effects of antibiotics on survival using bacteria-expressed Cry toxins than we observed in our studies with plant-expressed Cry toxins.

From our experiments, it is also clear that Bt plant variety significantly influenced survival as well as the interaction between Cry toxin and antibiotics, in particular for the species that exhibited moderate susceptibility, *S. littoralis*. Both non-Bt control maize varieties were fairly equally suitable as host plants for both lepidopteran species tested, with only minor differences in mortality, regardless of the antibiotic treatment—survival rates never dropped below 80%. However, the Spanish control maize variety was clearly less suitable for rapid weight gain and, thus, optimal development, than the South African maize variety, i.e. irrespective of the Cry toxin. This variety effect seemed to enhance the impact of the Cry toxins in the Spanish Bt maize variety decreasing the observed survival rate of *S. littoralis* larvae by almost 40% compared to the South African Bt maize. However, the antibiotic treatments buffered this impact significantly on Spanish Bt maize, allowing for about 15–20% higher survival than without antibiotics. This difference was also measurable but less distinct for *O. nubilalis* with much lower survival rates than *S. littoralis* on Bt maize. Maize plants are long known to produce insect deterring compounds like Dimboa or lignin (e.g., [35,36]) that likely may exert additional complex effects in combination with the expressed Cry toxins. While host plant resistance factors are well-known and have long been studied in many crop species, also in conjunction with other pest control measures such as pesticides (e.g., [37,38,39,40]), it represents an understudied field with regard to GM plants expressing insecticidal bacterial toxins [5,41].

### 3.2. Impact of Antibiotics on Efficacy of Cry1Ab Toxin from Bt Maize

Our data suggests that the expressed Cry toxins did not solely induce starvation (as cause of death) in the presence of antibiotics, i.e., presumed absence of microbiota, as hypothesized by Mason et al. [21]. For *O. nubilalis*, already on day 4, survival was reduced to less than 50% on both Bt maize varieties and for *S. littoralis* on the Spanish Bt maize to around 60%. In the study by Mason et al. [21], however, starvation did not affect survival rates before day 6. However, in our experiments, continuous presence of antibiotics or only a pretreatment with antibiotics until the onset of the bioassays did make some although small differences. This was possibly because even when stopping the administration of antibiotics at the beginning of the bioassay, a carry-over effect may last for the tested five-day period, meaning that the reestablishment of an effective gut microbiota probably takes longer than the testing period. Antibiotics alone did not affect larval survival on non-Bt control maize varieties, which is in agreement with all other studies listed in Table 1.

Interestingly, in our experiments with two different GM Bt maize varieties both expressing the Cry1Ab toxin from the same MON810 event, at least 15–20% of the *O. nubilalis* survived on both varieties. Although the effect of the antibiotic treatment was also pronounced in the Bt-spray treatment (Delfin) during the first four days, it was, however, lower than the reported levels of protection for various other lepidopteran pest larvae by Broderick et al. [19,20] and Mason et al. [21].

In conclusion, our results partially confirm findings of both sides of the contesting research groups [19,20,21,22,24,34] but they also differ in a number of aspects. From the published controversy, it seems that no party contests that gut microbiota play a significant role in the impact, or rather the unfolding of the impact of *B. thuringiensis* bacteria, spores and toxins on lepidopteran larvae. The core of the controversy centers around the question whether or not *B. thuringiensis* and its by-products are the main act or only have a support role—or, as Raymond et al. [23] have put it, whether *B. thuringiensis* is an ‘impotent pathogen’ or a full (killing) pathogen on its own right? With our studies, we cannot—and did not intend to—answer the question regarding the bacterial organism *B. thuringiensis.* However, we can answer the question regarding GM maize plants expressing solely more activated forms of the Cry toxin Cry1Ab. In our experiments, Cry toxins were the main act but with gut microbiota having a significant support role in conjunction with plant compounds. While the antibiotic treatment may have had an immune stimulating effect rendering the insects more tolerant to toxicemia, the lack of an effect in the survival or weight gain data of antibiotic treated larvae on control plants does not point to this effect. More research is needed, however, to reveal such underlying mechanisms further.

### 3.3. Implications

#### 3.3.1. For Efficacy of Bt Crops in Target Pests—Further Research Questions

In the field, organisms will encounter antibiotics only in situations when manure or slurry from industrial animal production systems are applied and where antibiotics are used on a regular basis and in large amounts [42,43]. However, the largest amounts of antibacterial substances are present in industrial agricultural production systems where GM plants with tolerance to glyphosate-based herbicides are grown. Glyphosate is known to have significant antibacterial effects [44] and to affect bacterial communities either in soils (e.g., [45]) or in intestines of mammals (e.g., [46,47,48]. Recently, researchers found that glyphosate significantly decreased the intestinal bacterial diversity also of an insect species, the honey bee (*Apis mellifera*) [49]. Roundup or other glyphosate-based herbicides are frequently applied in large amounts and, due to quickly evolving weed resistance against glyphosate-based herbicides, also in increasing concentrations. This applies to millions of hectares of arable land in North and South America and, to some extent, South Africa [4]. Incidentally, these production areas are identical to the areas where also Bt crops are grown at an industrial scale and, increasingly, these two GM traits (Bt and glyphosate tolerance), dominating global industrial agriculture since 20 years, are combined in so-called ‘stacked’ GM crop varieties. To our knowledge, no research has yet been conducted to study to what extent the antibacterial effect from the application of glyphosate-based herbicides may affect the efficacy of Bt toxins in GM Bt plants.

#### 3.3.2. For Ecotoxicity Testing of Bt Plants and Bt Insecticides

The documented impact of antibiotics on the efficacy of Cry toxins has important potential ramifications for ecotoxicity testing of nontarget organisms using artificial diets. In two recently published reports testing the ecotoxicity of Bt toxins on beneficial insects, high amounts of antibiotics were added to the test diets. While both Li et al. [28] and Ali et al. [29,30] based their recipes on those developed originally by Cohen and Smith [27], they deviated most significantly from the original recipe with regard to the addition of antibiotics. While Cohen and Smith [27] added antibiotics (streptomycin and chlortetracycline) at a concentration of 50 mg/100 g diet, Li et al. [28] more than tripled the amount to 180 mg/100 g diet (streptomycin and cephalosporin) and Ali et al. [29,30] even raised the concentration to an astounding 800 mg/100 g diet (streptomycin and penicillin). This significant deviation from the original recipe by Cohen and Smith [27] remains unexplained and unaccounted for. In contrast, Porcar et al. [50] who also studied nontarget effects of Bt toxins using artificial diets, clearly stated that “antibiotics were deliberately excluded from the diet composition since bacteria occurring in the insect midgut naturally might be critical for sensitivity“ [19], nipagin was chosen instead as preservative.

Also, van Frankenhuyzen [34] declared in a paper reporting about a study that addressed and expanded the experiments by Broderick et al. [19,20], that the 2 mg/mL total antibiotic used by Broderick et al. [19,20] as “high”. He further stated in that paper: “It is clear that the choice of antibiotics can profoundly affect experimental results” [34]. We would like to add that not only the choice but also the quantity of antibiotics will likely affect the outcome such as larval weight gain and survival.

## 4. Conclusions

We show that Cry toxins expressed in GM plants can act alone as a main killing agent in lepidopteran insects in the absence of gut bacteria, pointing to toxicemia being the main cause of death of the insect larvae. Furthermore, plant varietal differences in addition to differences in species sensitivity modify the toxicity of Cry toxins considerably. Additional investigations are needed to gain a clearer understanding of mechanistic details of the mode of action of Cry toxins, both of bacterial and plant origin, and how they are modulated by antibacterial compounds—not only in target pest species but in particular in non-target, beneficial organisms. Furthermore, artificial diets used for ecotoxicology testing and resistance evolution screening should be free of antibiotic compounds.

## 5. Materials and Methods

### 5.1. Insects

The eggs of *Spodoptera littoralis* were provided by Syngenta Crop Protection (Stein, Switzerland). The eggs of *Ostrinia nubilalis* were ordered from INRA (Nouvelle-Aquitaine-Poitiers, France). The larvae of both species were raised in a climate chamber under the following conditions: 16 h light, 8 h dark, 26 ± 1 °C and 60–70% relative humidity. The *S. littoralis* and *O. nubilalis* larvae were fed an artificial diet (based on Ivaldi-Sender [51], see Table 2) for three, respectively, five days.

Larvae receiving the antibiotics pre-treatment (in order to eliminate gut bacteria) were fed with an artificial diet amended with four broad-spectrum antibiotics. We used the same combination and concentrations of antibiotics as Broderick et al. [19,20]—the details are listed in Table 3.

### 5.2. Experimental Plants

The following four different maize varieties were used for the feeding bioassays: South African white Bt maize (variety: PAN6Q-321B, from Pannar) and a white non-Bt control maize (variety: PAN6Q-121, from Pannar), as well as Spanish yellow Bt maize (variety: PR33D48, from Pioneer/Du Pont) and a yellow non-Bt control maize variety (variety: DKC6666, from Dekalb/Monsanto). Both Bt maize varieties contained the same MON810 transgene construct expressing the Bt toxin Cry1Ab. The plants were grown in a climate chamber under the following identical conditions: 16 h light, 8 h dark, 20–25 °C, 50–65% relative humidity. Leaves were cut into smaller pieces (1.5 × 1.5 cm) and used for the bioassays. Leaves used for continuous antibiotics treatment were sprayed with antibiotics dissolved in distilled water and control leaves were sprayed with distilled water only. For each experiment, new antibiotic solution was prepared. The concentration of the antibiotics in the solution was the same as in the diet. Leaves treated with Bt pesticide were sprayed with Delfin^®^ (32,000 IU/mg) in concentration of 0.5 g/L, as recommended by the manufacturer (Syngenta).

### 5.3. Midgut Bacterial Strains and Culture Conditions

Traditional culture-methods used were applied to study midgut microbiota of insect larvae exposed to antibiotics and fed MON810 GM maize. Larvae midguts were harvested and grinded with glass balls, and successive dilutions of the supernatant (10^−0^ to 10^−6^, owing to obtain 30 to 300 CFU per Petri dish) were incubated in selective agar media (Table 4) during 72 h; all colonies were counted after 24, 48, or 72 h depending on the medium (see Table 4). Experiments were performed in triplicate.

### 5.4. Bioassays

After raising *S. littoralis* and *O. nubilalis* larvae on the artificial diet with or without antibiotics for three and five days respectively, the larvae were weighed and placed individually into bioassay trays with 32 cells, each cell containing a moist filter paper and a piece of maize leaf. The experimental set up is shown in Table 5. The experiment was repeated three times with *S. littoralis* and four times with *O. nubilalis.*

For *S. littoralis*, the weight was recorded for groups of eight larvae prior to the bioassay on day 0 and for the surviving larvae from the same group on day 5. Weight gain was calculated by subtracting the initial larval weight on day 0 from the final weight on day 5.

For *O. nubilalis*, the weight was recorded only for a subset of larvae prior to the bioassay on day 0 in order to check for any possible effects of the antibiotics on the larvae weight. There were no significant differences in the weight of larvae fed with or without antibiotics (data not shown).

Survival of the larvae was recorded on days 3, 4, and 5 after setting up the bioassay. The leaf pieces had been replaced on day 3 of the experiment and the filter papers had been moistened daily. The bioassays were carried out in the climate chamber under the following conditions: 16 h light, 8 h dark, 26 ± 1 °C and 60–70% relative humidity.

### 5.5. Statistics

Statistical analyses were performed with the R software, version 3.2.3 and Systat, version 13. The binomial response variable ‘survival’ (i.e., probability or proportion of survival) was analyzed with Cox’s proportional hazard [54] test (‘coxph’ function) from the ‘survival’ package in R. We used data for animals that were dead at day 3, 4, and 5 and censored the data for individual insects that were alive on day 5. We used the function *survfit* to model expected survival for all groups, with 95% confidence intervals. These models were used for graphical representation of survival within each treatment group.

Larval weight from the feeding bioassay was tested with analyses of covariance (ANCOVA). We used log transformed ‘weight gain’ as a dependent variable and ‘plant type’ (Bt and non-Bt), ‘antibiotic treatment’ (three or five days’ pretreatment, continuous, and no treatment). In addition, we had three and four independent replications (*S. littoralis* and *O. nubilalis*, respectively) for the feeding bioassay which were used as a covariate in the analyses.

## Figures and Tables

**Table 1:** Summary of suggested and contested modes of action behind the observed interactions of Bt proteins with gut bacteria.

Reference	Route of Administration, Type of Bt Toxin		Source of Bt Toxins	Test Species, Tested Lifestage	Treatments	Observed Effects (% Survival)
	Antibiotics	Bt toxins				
Broderick et al. 2006 [19]	AD	Cry1Aa, Cry1Ab, Cry1Ac, Cry2A proteins (crystals) and spores; Cry1Aa protoxins	Dipel, commercial formulation of *B. thuringiensis* var. *kurstaki* incl. other compounds for formulation; *E. coli* strain ECE52 producing	*Lymantria dispar* L3	Pre-treatment AB prior to assay: Rifampin, Gentamicin, Penicillin, Streptomycin; Gut bacteria: *Enterobacter* sp. NAB3, *Enterococcus casseliflavus*, *Staphylococcus xylosus*;	Close to zero survival without antibiotics, 100% survival with no gut bacteria present (with antibiotics) after 7 days. No timeline for survival **Hypothesis:** *Septicemia by gut bacteria Cry toxins enable gut bacteria to reach hemocoel (breaching gut wall) but does not kill the larvae.*
Broderick et al. 2009 [20]	AD	Cry1Aa, Cry1Ab, Cry1Ac, Cry2A proteins (crystals) and spores Cry1Ac protoxins encapsulated in *Pseudomonas fluorescens*	Dipel, commercial formulation of *B. thuringiensis* var. *kurstaki* incl. other compounds for formulation; MPVII, commercial formulation incl. other compounds for formulation	*Vanessa cardui*, *Manduca sexta*, *Heliothis virescence* L3 *Pieris rapae* L4 *Pectinophorella gossypiella*, *Lymantria dispar* L?	Pre-treatment AB prior to assay: Rifampin, Gentamicin, Penicillin, Streptomycin; Gut bacteria: *Enterobacter* sp. NAB3	For all, except *P. gossypiella*, survival exceeded 90% in presence of antibiotics after 7 days. For *P. gossypiella* another mechanism seemed at work No timeline of survival given but mentioned that “antibiotics greatly delayed time ... to kill.” **Hypothesis:** Except for *P. gossypiella*, **septicemia** caused by *Enterobacter* and not Bt—confirmed by use of MPVII free Bt and live cells
Mason et al. 2011 [21]	AD, injections	Cry1Ac protoxins encapsulated in *Pseudomonas fluorescens*	MPVII, commercial formulation incl. other compounds for formulation	*Manduca sexta*, L5	Pre-treatment AB prior to assay: rifampin, gentamicin, nystatin (antifungal); Gut bacteria: *Enterococcus faecalis* (Ef) ± Bt; starvation	*E. faecalis* + Bt—0% at day 5 Bt 0% at day 8 (like starvation)—delay in mortality—although no time line presented *Hypothesis*: *E. faecalis* induced **septicemia**, Bt toxin caused paralysis and death due to starvation—**combined effect** *E. faecalis* + Bt
Raymond et al. 2009 [22]	AD	Cry 1 and Cry 2 crystals Cry1Ac crystals	HD-1 strain derived from Dipel, commercial formulation of *B. thuringiensis* var. *kurstaki* HD-73, and rifampicin resistant strains of HG-73 reif^R^	*Plutella xylostella* L2 and L3	Pre-treatment AB prior to assay: Rifampin Gut bacteria: *Enterobacter* sp. Mn2	Significant delays in mortality onset with ABs 0% survival when Bt w/o AB or AB-resistant Bt was used Cry1Ac from HD-73 survival 10% w/o AB, 20% with AB, effect annulled when AB-resistant HD-73 strain was used *Hypothesis*: **Septicemia by Bt and toxins**, **no gut bacteria necessary** to kill larvae.
Johnston and Crickmore 2009 [24]	AD	Cry 1 and Cry 2 toxins crystals Cry1Ac crystals	HD-1 strain derived from Dipel, commercial formulation of *B. thuringiensis* var. *kurstaki* HD-73, and rifampicin resistant strains of HG-73 reif ^R^	*Manduca sexta* L2 and L3	Pre-treatment AB prior to assay: first instar only or continuously	Timelines show significant delays in mortality onset with ABs Cry1Ac from HD-73: 0% survival w/o AB, 10% with AB after 6 days Dipel: 0% survival w/o AB, 20% survival with AB after 6 days *Hypothesis*: **Septicemia by Bt and toxins**, **no gut bacteria**

AB antibiotics; AD artificial diet for lepidopteran larvae; ? undetermined; [21]—Bt toxin still must form pores so that *E. faecalis* can translocate into hemocoel.

**Table 2:** Detailed information of ingredients of the artificial diet.

Ingredient	Amount	Ingredient Description	Manufacturer
**Total Volume**	0.5 L	-	-
**H_2_O Dest.**	390 mL	-	-
**Agar**	10 g	-	Sigma-Aldrich (Buchs, S)
**Maize Semolina**	25 g	M Classic Polenta 2 min, Maisgriess	Migros (Zurich, S)
**Wheat Germ**	25 g	Qualité & Prix Weizenkeime	Coop (Zurich, S)
**Yeast Powder**	25 g	Actilife Vitamin-Bierhefe	Migros (Zurich, S)
**Benzoic Acid**	0.9 g	-	Sigma-Aldrich (Buchs, S)
**Nipagin**	0.9 g	Methyl 4-hydroxybenzoate	Sigma-Aldrich (Buchs, S)
**Ascorbic Acid**	2.25 g	L-ascorbic acid	Sigma-Aldrich (Buchs, S)

S: Switzerland.

**Table 3:** Details of antibiotics added to artificial diet.

Ingredient	Amount	Ingredient Description	Manufacturer
**Penicillin G Potassium Salt**	500 mg/L	~1600 units/mg	Sigma-Aldrich (Buchs, S)
**Gentamycin Sulfate**	500 mg/L	From *Micromonospora purpurea*, −700 U/mg dried material	Sigma-Aldrich (Buchs, S)
**Rifampicin**	500 mg/L	≥97% (HPLC) powder	Sigma-Aldrich (Buchs, S)
**Streptomycin Sulfate Salt**	500 mg/L	-	Sigma-Aldrich (Buchs, S)

S: Switzerland.

**Table 4:** Media and conditions for strains isolation and culture (based on Poulsen et al. [52] and Muñoa and Pares [53]).

Selection	Media	Conditions
	Agar plates	
**Total Aerobes**	Plate Count Agar (Biokar, Beauvais, France)	72 h Aerobically, 30 °C
**Total Anaerobes**	Plate Count Agar (Biokar, Beauvais, France)	72 h Anaerobically (AnaeroGen 2.5 L, Sigma-Aldrich), 30 °C
**Enterococci**	Slanetz and Bartley (Biokar, Beauvais, France)	48 h Aerobically, 37 °C

**Table 5:** The 12 bioassays. Ab 3/5 days: Larvae were reared for three days (*S. littoralis*) or five days (*O. nubilalis*) on the antibiotics amended diet prior to bioassay. Ab cont.: Larvae were reared for 3/5 days on the antibiotics amended diet prior to bioassay. During the bioassay, the antibiotic solution was sprayed on the maize leaves on day 0 and 3. 0 Ab (Control): Larvae were raised without antibiotics.

Maize Varieties	Antibiotic Treatment (Ab)		
	Ab 3/5 days	Ab Cont.	0 Ab (Control)
**Bt South Africa**	32 larvae	32 larvae	32 larvae
**non-Bt South Africa**	32 larvae	32 larvae	32 larvae
**Bt Spain**	32 larvae	32 larvae	32 larvae
**non-Bt Spain**	32 larvae	32 larvae	32 larvae
